# Supplementary material for: Similar neuronal imprint and no cross-seeded fibrils in α-synuclein aggregates from MSA and Parkinson’s disease
Source: NPJ Parkinsons Dis. 2022 Jan 13;8:10. doi: 10.1038/s41531-021-00264-w (PMC8758785; doi:10.1038/s41531-021-00264-w)
Supplement: Supplementary file 1 — REPORTING SUMMARY [file 41531_2021_264_MOESM1_ESM.pdf]

nature portfolio

Corresponding author(s): Florent LAFERRIERE
François ICHAS

Last updated by author(s): Oct 12, 2021

Reporting Summary

Nature Portfolio wishes to improve the reproducibility of the work that we publish. This form provides structure for consistency and transparency in reporting. For further information on Nature Portfolio policies, see our Editorial Policies and the Editorial Policy Checklist.

Statistics

For all statistical analyses, confirm that the following items are present in the figure legend, table legend, main text, or Methods section.

n/a	Confirmed	
☐	☒	The exact sample size (*n*) for each experimental group/condition, given as a discrete number and unit of measurement
☐	☒	A statement on whether measurements were taken from distinct samples or whether the same sample was measured repeatedly
☐	☒	The statistical test(s) used AND whether they are one- or two-sided<br>*Only common tests should be described solely by name; describe more complex techniques in the Methods section.*
☐	☒	A description of all covariates tested
☐	☒	A description of any assumptions or corrections, such as tests of normality and adjustment for multiple comparisons
☐	☒	A full description of the statistical parameters including central tendency (e.g. means) or other basic estimates (e.g. regression coefficient) AND variation (e.g. standard deviation) or associated estimates of uncertainty (e.g. confidence intervals)
☐	☒	For null hypothesis testing, the test statistic (e.g. *F*, *t*, *r*) with confidence intervals, effect sizes, degrees of freedom and *P* value noted<br>*Give P values as exact values whenever suitable.*
☒	☐	For Bayesian analysis, information on the choice of priors and Markov chain Monte Carlo settings
☒	☐	For hierarchical and complex designs, identification of the appropriate level for tests and full reporting of outcomes
☐	☒	Estimates of effect sizes (e.g. Cohen's *d*, Pearson's *r*), indicating how they were calculated

Our web collection on statistics for biologists contains articles on many of the points above.

Software and code

Policy information about availability of computer code

Data collection	Immunoblot chemiluminescence: Chemidoc Station (Biorad)<br>Proteomics: Xcalibur 4.1
Data analysis	Statistics: Prism 9.0 (GraphPad)<br>Immunoblot: Image Lab (Biorad), Image Studio Lite 4.0.21 (LI-COR)<br>Proteomics: Proteome Discoverer 2.3 (Thermo Fisher Scientific Inc.) and Panther Gene Ontology (http://pantherdb.org)

For manuscripts utilizing custom algorithms or software that are central to the research but not yet described in published literature, software must be made available to editors and reviewers. We strongly encourage code deposition in a community repository (e.g. GitHub). See the Nature Portfolio guidelines for submitting code & software for further information.

Data

Policy information about availability of data

All manuscripts must include a data availability statement. This statement should provide the following information, where applicable:

- Accession codes, unique identifiers, or web links for publicly available datasets
- A description of any restrictions on data availability
- For clinical datasets or third party data, please ensure that the statement adheres to our policy

Mass spectrometry raw datasets were uploaded to the ProteomeXchange Consortium via the PRIDE partner repository. Dataset identifier: PXD024998.
Data availability : The data that support the findings of this study are available from the corresponding author upon request (postmortem human brain samples used in this study are unique biological samples and are therefore not available on request, but similar samples – disease, brain region, age, gender – can be obtained upon adequate request to any official brain bank.

Field-specific reporting

Please select the one below that is the best fit for your research. If you are not sure, read the appropriate sections before making your selection.

☒ Life sciences ☐ Behavioural & social sciences ☐ Ecological, evolutionary & environmental sciences

For a reference copy of the document with all sections, see nature.com/documents/nr-reporting-summary-flat.pdf

Life sciences study design

All studies must disclose on these points even when the disclosure is negative.

Sample size	No statistical methods were used to pre-determine sample sizes but our sample sizes are similar to those reported in previous publications cited in main text.
Data exclusions	No data were excluded from our analysis.
Replication	All experiments represented in the study were repeated as mentioned in Methods section and figure legends.<br>Immunoblots and ultracentrifugation gradient replication consisted of an experimentally independent repetition with same biological samples, as well as performing the same experiment with all biologically independent samples indicated.<br>All attempts for replication were successful. Patient variability is detailed in our manuscript.
Randomization	Patient samples were classified into disease subtypes, based on the clinical, genetic and neuropathological analysis done at the respective site, where the autopsies were performed.<br>Data collection was randomized as control / disease patient samples were assigned randomly to experimental procedures. We also ensured that all different sample groups were included in each experiment to exclude any experiment / experimenter biases. Similarly, in all SarkoSpin, mass spectrometry, ultracentrifugation and immunolabelling procedures, all synucleinopathies samples were randomized with control samples.
Blinding	Data collection and analysis were not performed blind to the conditions of the experiments as they did not include any subjective measurement or incomplete data collection.

Reporting for specific materials, systems and methods

We require information from authors about some types of materials, experimental systems and methods used in many studies. Here, indicate whether each material, system or method listed is relevant to your study. If you are not sure if a list item applies to your research, read the appropriate section before selecting a response.

Materials & experimental systems

n/a	Involved in the study
☐	☒ Antibodies
☒	☐ Eukaryotic cell lines
☒	☐ Palaeontology and archaeology
☒	☐ Animals and other organisms
☒	☐ Human research participants
☒	☐ Clinical data
☒	☐ Dual use research of concern

Methods

n/a	Involved in the study
☒	☐ ChIP-seq
☒	☐ Flow cytometry
☒	☐ MRI-based neuroimaging

Antibodies

Antibodies used	Antibody Target Company Cat.No Dilution IB<br><br>Primary antibodies<br>MJFR-1 human alpha-synuclein Abcam ab138501 1 : 10,000<br>EP1536Y pS129 phospho-synuclein Abcam ab51253 1 : 5,000<br>LB509 aggregated alpha-synuclein Biolegend 807701 1 : 2,000<br>Actin beta-actin Sigma A5316 1 : 10,000<br>Ferritin ferritin H + L Abcam ab75973 1 : 5,000<br>Arginase arginase-1 Abcam ab133543 1 : 1,000<br>ATP8A1 phospholipid-transporting ATPase IA Sigma HPA052935 1 : 1,000<br>Calnexin calnexin Abcam ab22595 1 : 1,000<br>Cyclophilin B peptidyl-prolyl cis-trans isomerase B Abcam ab178397 1 : 1,000<br>Fumarase fumarate hydratase Abcam ab233394 1 : 1,000<br>GluT3 solute carrier 2, glucose transporter 3 Abcam ab191071 1 : 1,000<br>NipSnap-1 protein nipsnap homolog 1 Sigma HPA059227 1 : 1,000

PME-1 protein phosphatase methylesterase 1 Abcam ab205956 1 : 1,000
Septin-5 septin-5 Sigma SAB1401289 1 : 1,000
SFXN1 sidoreflexin 1 Sigma HPA019543 1 : 1,000
Ferritin ferritin H + L chains Abcam ab75973 1 : 5,000
Actin beta-actin Sigma A5316 1 : 10,000

Secondary antibodies
Goat anti-mouse HRP mouse IgG (H+L) Jackson Immuno 115-035-146 1 : 10,000
Goat anti-rabbit HRP rabbit IgG (H+L) Jackson Immuno 111-035-144 1 : 10,000
Goat anti-mouse IRDye 680 mouse IgG (H+L) LI-COR 926-68070 1 : 5,000
Goat anti-rabbit IRDye 800 rabbit IgG (H+L) LI-COR 926-32211 1 : 5,000

Validation

Each antibody was validated by the correspondent manufacturer, and is publicly available on its website with indicated catalogue numbers. Antibodies against proteomics candidates were also validated by western blots as shown in the supplementals section.
