# Supplementary material for: Similar neuronal imprint and no cross-seeded fibrils in α-synuclein aggregates from MSA and Parkinson’s disease
Source: NPJ Parkinsons Dis. 2022 Jan 13;8:10. doi: 10.1038/s41531-021-00264-w (PMC8758785; doi:10.1038/s41531-021-00264-w)
Supplement: Supplementary file 2 — Supplementary Information [file 41531_2021_264_MOESM2_ESM.pdf]

SUPPLEMENTARY INFORMATION

SUPPLEMENTARY FIGURES

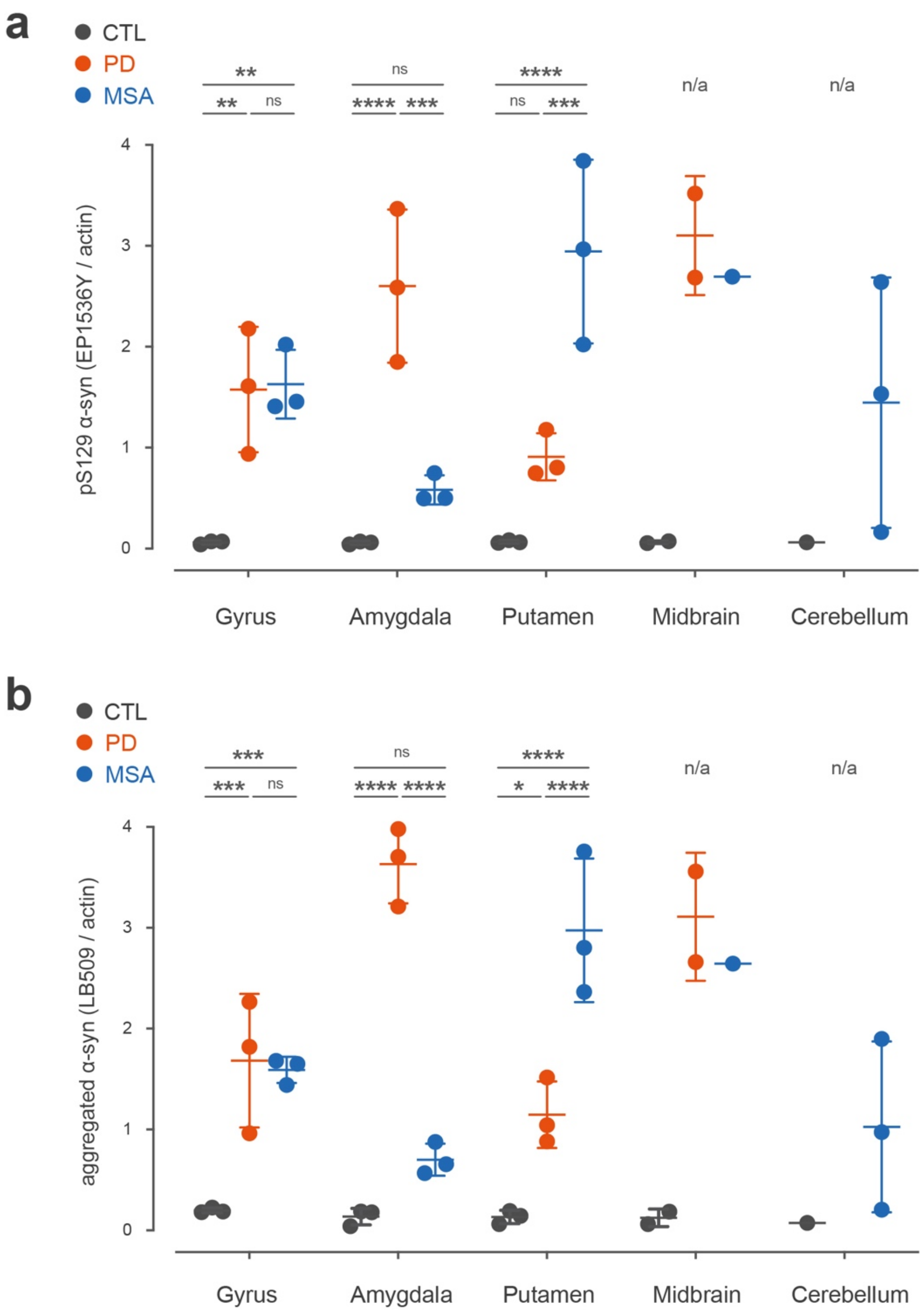

Supplementary Figure 1. Comparison of pathological alpha-synuclein load in different brain regions of synucleinopathy subjects by immunoblotting. Brain homogenates from different brain regions (gyrus, amygdala, putamen, midbrain and cerebellum) of the three control (black), PD (red) and MSA (blue) human subjects of the study were subjected to dot blot and subsequent immunolabelling and quantification of pS129-positive (**a**, EP1536Y/actin) and aggregated (**b**, LB509/actin) α-synuclein load. The relative amounts (A.U.) are plotted for each subject brain region, and the respective p-values of Tuckey corrected two-way ANOVAs are represented above each couple of comparisons.

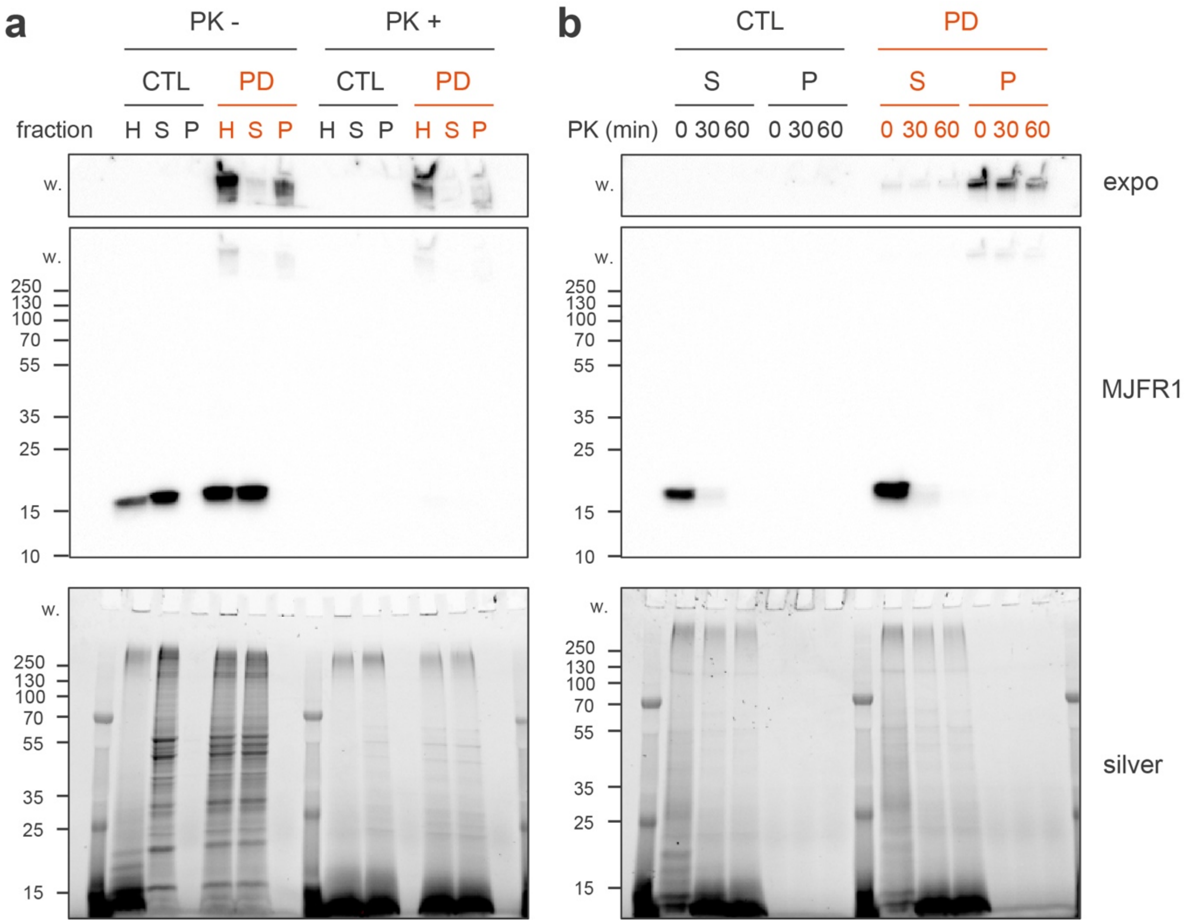

Supplementary Figure 2. Enrichment in SDS-insoluble, denaturation- PK-resistant high molecular weight alpha-synuclein species in Sarkospin pellets of synucleinopathy brain samples. Biochemical analysis of SDS- and PK-resistance of α-synuclein species contained in Sarkospin fractions extracted from human brain samples. Total solubilized homogenates (H), Sarkospin supernatants (S) and pellets (P) were treated or not for 1 hour (PK+/-, **a**) or treated for the indicated time (0-60 min, **b**) with 1 μg.ml^{-1} PK at 37°C prior to be denatured in Laemmli at 95°C for 5 min, subjected to SDS-PAGE and stained for total proteins (silver) or immunoblotted against human α-synuclein (MJFR1) after transfer on nitrocellulose membrane and fixation. w. indicates the wells and stacking gels were high molecular weight SDS-resistant proteins are retained. Increased signal exposure of these part of membranes are represented in separated boxes (expo.) for better visualization of the latter HMW species.

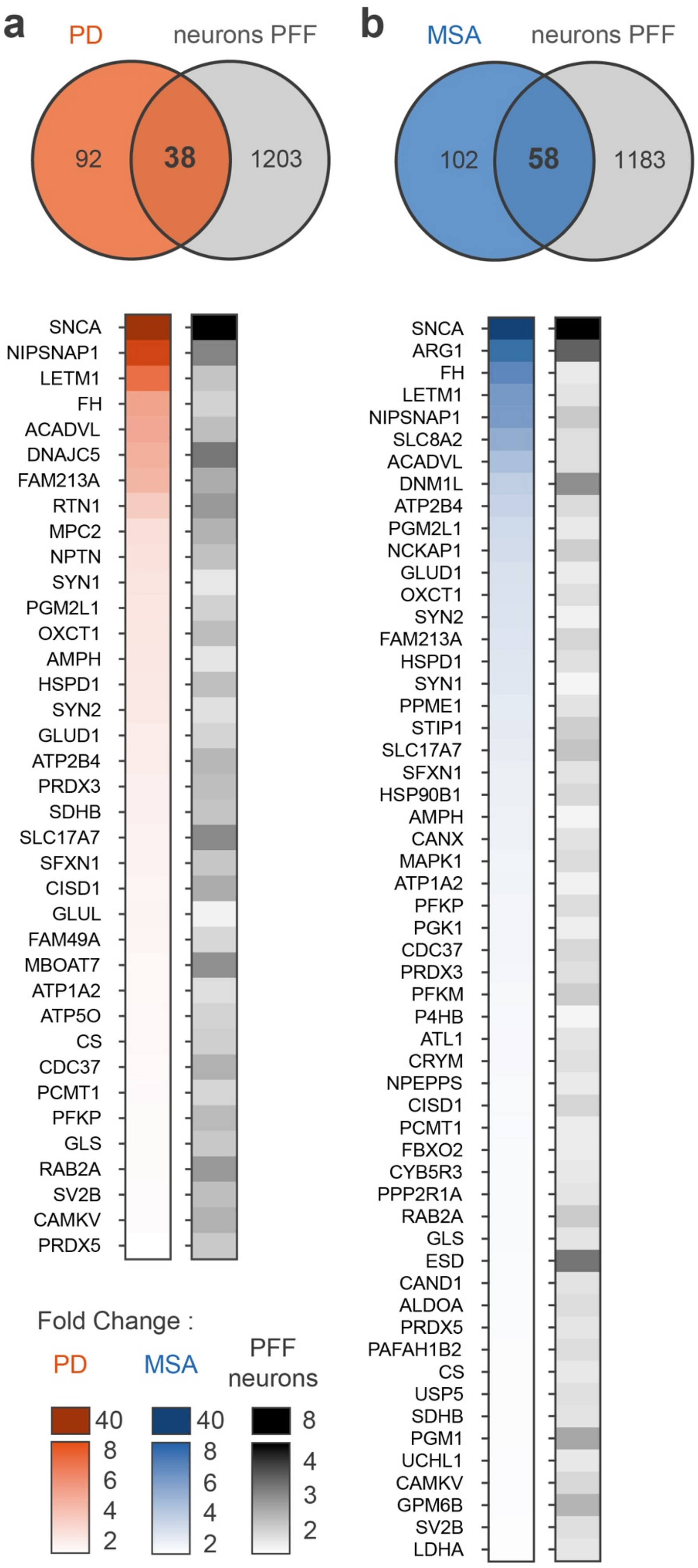
a
PD
neurons PFF
92
38
1203
b
MSA
neurons PFF
102
58
1183
SNCA
NIPSNAP1
LETM1
FH
ACADVL
DNAJC5
FAM213A
RTN1
MPC2
NPTN
SYN1
PGM2L1
OXCT1
AMPH
HSPD1
SYN2
GLUD1
ATP2B4
PRDX3
SDHB
SLC17A7
SFXN1
CISD1
GLUL
FAM49A
MBOAT7
ATP1A2
ATP5O
CS
CDC37
PCMT1
PFKP
GLS
RAB2A
SV2B
CAMKV
PRDX5
SNCA
ARG1
FH
LETM1
NIPSNAP1
SLC8A2
ACADVL
DNM1L
ATP2B4
PGM2L1
NCKAP1
GLUD1
OXCT1
SYN2
FAM213A
HSPD1
SYN1
PPME1
STIP1
SLC17A7
SFXN1
HSP90B1
AMPH
CANX
MAPK1
ATP1A2
PFKP
PGK1
CDC37
PRDX3
PFKM
P4HB
ATL1
CRYM
NPEPPS
CISD1
PCMT1
FBXO2
CYB5R3
PPP2R1A
RAB2A
GLS
ESD
CAND1
ALDOA
PRDX5
PAFAH1B2
CS
USP5
SDHB
PGM1
UCHL1
CAMKV
GPM6B
SV2B
LDHA
Fold Change :
PD
MSA
PFF neurons
40
8
6
4
2
40
8
6
4
2
8
4
3
2

Supplementary Figure 3. Analysis of the overlaps of insoluble proteomes from synucleinopathy brain samples and α-syn PFF-treated mouse primary neurons[12].

The lists of gene names corresponding to the 130 and 160 proteins enriched in our proteomic study in PD (**a**) and MSA (**b**) pellets, respectively, were searched against the list of gene names of the 1241 unique proteins significantly enriched in insoluble pellets from mouse primary neurons at 14 and 21 dpi treated with recombinant α-syn PFF in Mahul-Mellier et al., 2020[12]. Venn diagrams show that 38 and 58 proteins are common to PD and MSA pellets and PFF-treated neurons insoluble proteins. These proteins are represented in the diagram below, with color-coding their fold change of enrichment PD (red) or MSA (blue) vs CTL, together with their respective mean (14 - 21 dpi) fold change in PFF vs PBS treated mouse primary neurons (grayscale).

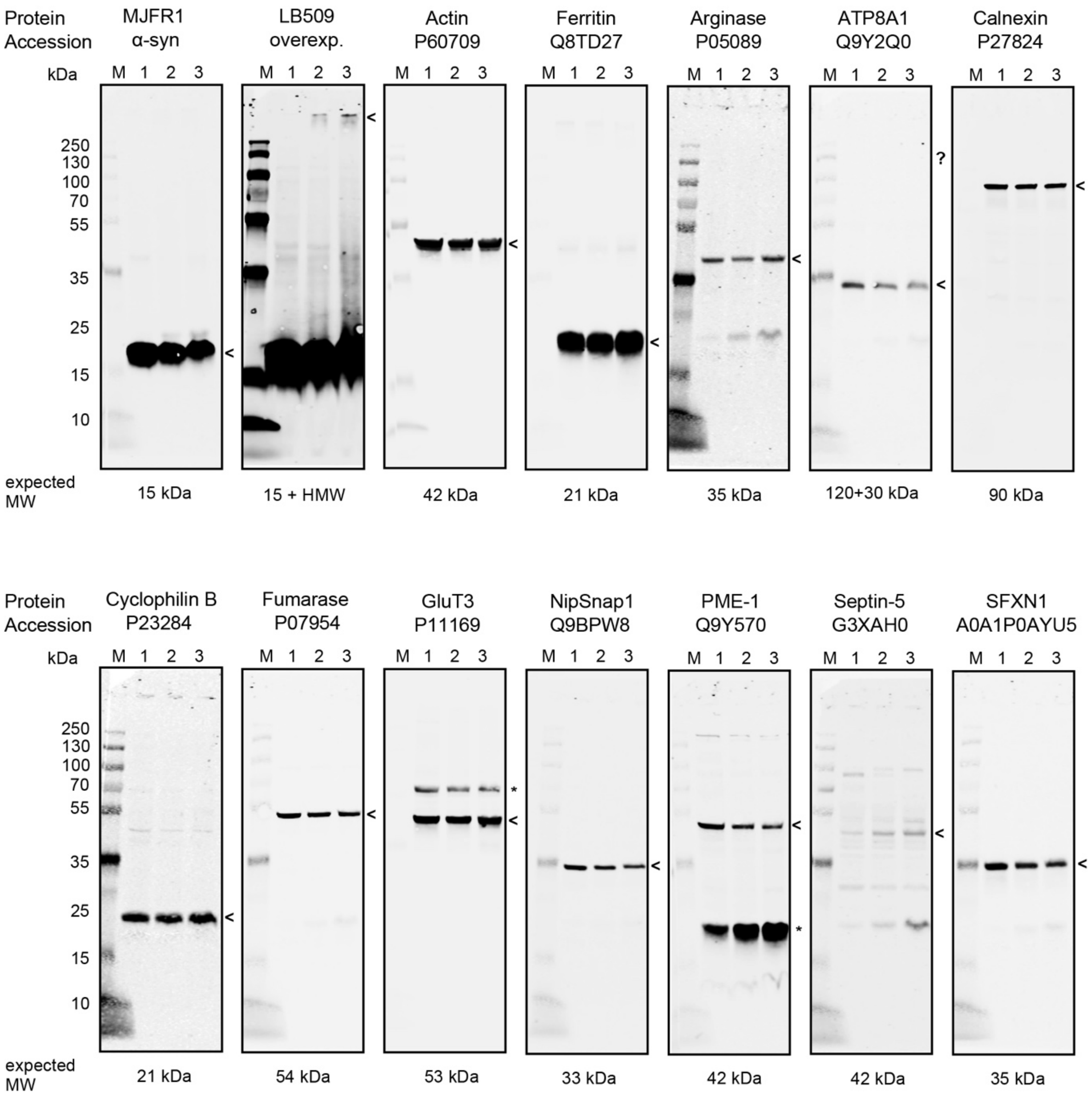

Supplementary Figure 4. Validation of the selectivity of antibodies directed to candidates of interest by SDS-PAGE. Brain homogenates pools from n=3 controls (lanes 1), PD (lanes 2) and MSA (lanes 3) gyrus samples were denatured by boiling in Laemmli, subjected to SDS-PAGE and blotted on nitrocellulose (lanes M = MW marker). Immunolabelling with antibodies directed to the indicated targets showed bands at the expected size (expected MW, indicated by arrowheads). Stars are pointing at unspecific signals for PME-1, ATP8A1 and GluT3.

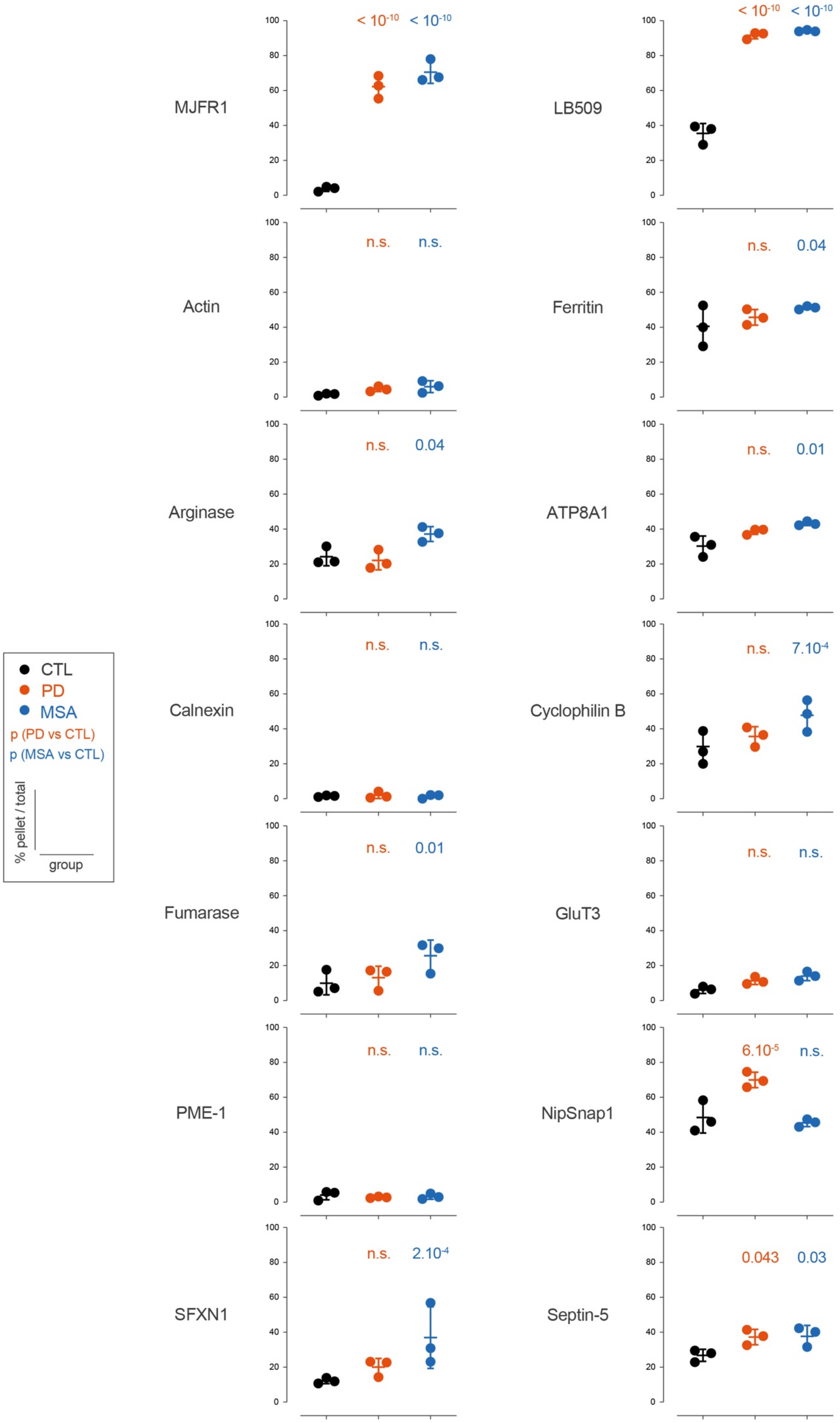

MJFR1
$< 10^{-10}$
$< 10^{-10}$
LB509
$< 10^{-10}$
$< 10^{-10}$
Actin
n.s.
n.s.
Ferritin
n.s.
0.04
Arginase
n.s.
0.04
ATP8A1
n.s.
0.01
Calnexin
n.s.
n.s.
Cyclophilin B
n.s.
7.10^{-4}
Fumarase
n.s.
0.01
GluT3
n.s.
n.s.
PME-1
n.s.
n.s.
NipSnap1
6.10^{-5}
n.s.
SFXN1
n.s.
2.10^{-4}
Septin-5
0.043
0.03
0
20
40
60
80
100
CTL
PD
MSA
p (PD vs CTL)
p (MSA vs CTL)
% pellet / total
group

Supplementary Figure 5. Validation of the enrichment of candidates of interest by dot blot on Sarkospin fractions. Brain homogenates from n=3 controls (black), PD (red) and MSA (blue) gyrus samples were subjected to Sarkospin fractionation. Supernatant and pellet fractions were loaded on drop blot (left) and filter trap (right) and immunolabelled with antibodies directed against the indicated proteins. Each protein amount was quantified in Sarkospin fractions and is plotted as relative pelleted amount (% pellet/total). P-values of the corresponding Tuckey-corrected two-ways ANOVAs of the comparison of insolubility of each target for PD (red) or MSA (blue) vs control, respectively, are shown above the plots. MJFR1 (total α-syn), LB509 (aggregated α-syn), actin and ferritin are shown as controls. PME-1, GluT3 and Calnexin were not validated using these techniques, possibly because of their weak immunodetection, the presence of unspecific bands or isoforms (Supplementary Fig. 4), or to the inaccessibility of the epitopes in native conditions of dot blotting.

DATASETS

DataSet 1. List of all proteins detected by mass spectrometry in any Sarkospin pellet.

DataSet 2. List of 130 proteins enriched in PD.

DataSet 3. List of 160 proteins enriched in MSA.

DataSet 4. List of 206 unique proteins gated as enriched in PD and/or MSA.

DataSet 5. List of 84 gated proteins enriched in both PD and MSA.

DataSet 6. List of 3 gated proteins specifically enriched only in PD.

DataSet 7. List of 4 gated proteins specifically enriched only in MSA.

DataSet 8. List of gene ontology clusters statistically overrepresented in PD and MSA samples.
